# The Role of Untraceable Sentinel Lymph Nodes in Prostate Cancer Patients Undergoing Radical Prostatectomy and Pelvic Lymph Node Dissection: Insights from an Ongoing Prospective Study

**DOI:** 10.3390/jcm14248852

**Published:** 2025-12-15

**Authors:** Zilvinas Venclovas, Donatas Vajauskas, Paulius Jarusevicius, Gustas Sasnauskas, Tomas Ruzgas, Mindaugas Jievaltas, Daimantas Milonas

**Affiliations:** 1 Department of Urology, Lithuanian University of Health Sciences, LT-44307 Kaunas, Lithuania; 2 Department of Radiology, Lithuanian University of Health Sciences, LT-44307 Kaunas, Lithuania; 3 Department of Applied Mathematics, Kaunas University of Technology, LT-44249 Kaunas, Lithuania

**Keywords:** prostate cancer, radical prostatectomy, sentinel lymph node

## Abstract

**Background/Objectives:** The role of extended pelvic lymph node dissection (ePLND) in prostate cancer remains uncertain. Sentinel lymph node (sLN) mapping improves diagnostic precision, yet some patients have no detectable sentinel nodes (“untraceable” sLNs). This study evaluates whether untraceable sLNs predict the absence of lymph node invasion (LNI) and can guide surgical decision-making during radical prostatectomy (RP) with ePLND. **Methods:** Patients with intermediate- or high-risk prostate cancer and with no radiologically evident LNI were included in the study. A ^99m^Tc-nanocolloid was used as an sLN tracer. RP with sLN dissection and ePLND was performed <20 h after injection. Patients were categorized into two groups: Group 1, traceable sLNs and Group 2, untraceable sLNs (no radiological or intraoperative signal). **Results:** A total of 53 patients were included. LNI was present in 10 patients (18.9%). Group 1 had 41 patients (77.4%), and Group 2 had 12 patients (22.6%). None of the patients in Group 2 had LNI following ePLND, whereas 10 of 41 patients (24.4%) in Group 1 were node-positive (*p* = 0.016). Baseline clinical and pathological characteristics were comparable between groups. A total of 17/53 of men (32.1%) experienced biochemical recurrence, overall, with higher observed events in Group 1 (15/41, 36.6%) vs. Group 2 (2/12, 16.7%). However, this difference did not reach statistical significance (*p* = 0.2). **Conclusions:** A proportion of PCa patients have no radiologically or intraoperatively detectable sLNs, and none of the patients with untraceable sLNs exhibited LNI following ePLND. These findings suggest that untraceable sLNs may correlate with an extremely low probability of nodal invasion and could serve as a criterion for safely omitting ePLND in selected patients.

## 1. Introduction

According to global cancer statistics, prostate cancer (PCa) remains one of the most frequently diagnosed malignancies in men. Although mortality is decreasing, in 2022, approximately 1.4 million new cases were diagnosed, and almost 400 thousand deaths were documented worldwide [1]. Several PCa treatment options are available; however, radical prostatectomy (RP) remains one of the most commonly used approaches [2].

Pelvic lymph node dissection (PLND) performed during RP is the most accurate staging procedure for the detection of lymph node invasion (LNI) in PCa [3]. The detection of LNI at PLND can be as high as 25% and is directly associated with the extent of PLND and the aggressiveness of PCa [4,5,6,7,8]. No doubt, extended PLND (ePLND) provides more accurate staging in comparison with limited PLND (lPLND), and it has recently been recommended as a standard procedure by most international urological guidelines [2,9,10]. However, ePLND is associated with longer duration of general anesthesia and surgery, as well as higher rates of postoperative complications, compared with lPLND [11].

The indications for PLND continue to be controversial. It is proven that ePLND demonstrates the benefit of disease staging, but there is no definitive evidence that it confers an oncological survival benefit, and randomized controlled trials are lacking [2,12]. Consequently, some authors have advocated the use of sentinel lymph node (sLN) dissection with ^99m^Tc-nanocolloid. This technique is already used in daily practice for melanoma, breast, penile and vulvar cancers [13]. Although the role of sLN dissection in prostate cancer is still under debate, sentinel lymphadenectomy is accurate for nodal staging with high sensitivity, specificity, positive predictive value (PPV) and negative predictive value (NPV) [2,13,14]. sLN mapping improves diagnostic precision, yet some subset of patients have no detectable sentinel nodes with no radiological or intraoperative signal (“untraceable” sLNs).

This study prospectively evaluates whether untraceable sLNs predict the absence of LNI and whether this information can guide intraoperative decision-making regarding RP with ePLND.

## 2. Materials and Methods

In this prospective study, we consecutively included 53 patients treated at the Department of Urology, Lithuanian University of Health Sciences, between January 2021 and September 2025, who underwent sLN dissection followed by ePLND and open radical prostatectomy. Patients with localized PCa and an MSKCC nomogram [15]-assessed risk of ≥7% for lymph node involvement were eligible for inclusion. All men without suspicious lymph nodes on the chest–abdomen–pelvic computer tomography (CT), bone scan or pelvic magnetic resonance imaging (MRI), were considered candidates for the sLN dissection. During the final year of the study, PET/PSMA became available at our institution and was additionally used in combination with other radiological tests.

Preoperative data included age, clinical stage (cT), preoperative PSA, biopsy ISUP, percentage of positive biopsy cores, percentage of MSKCC nomogram, body mass index (BMI) and activation of the ^99m^Tc-nanocolloid. Pathological stage (pT), positive surgical margins status (R1), pathological ISUP, number of removed lymph nodes and lymph node invasion (LNI) were registered after RP. Pathological stage was assessed using the 2002 TNM system, and tumor grading was classified by using the 2014 ISUP suggested grade grouping [16].

The ^99m^Tc-nanocolloid was used as the radiotracer. The injection was performed the day before the surgery (less than 20 h preoperatively). It was guided with transrectal ultrasound, punctured using a Chiba needle (0.95 mm × 220 mm) in the peripheral zone of both lobes and distributed into the four quadrants. All patients had antibiotic prophylaxis with 500 mg of cefuroxime orally before and after the procedure. One hour before surgery, an abdominal–pelvic SPECT/CT scan was performed for radiological identification of sLNs.

During open radical prostatectomy and in order to validate the technique, ePLND was performed in addition to sLN dissection. The ePLND included at least the following nodal regions: external and internal iliac LNs, obturator LNs and common iliac LNs up to the crossing of the ureters. Each nodal packet was sent separately for pathological analysis and data collection.

An sLN was defined as the lymphatic tissue expressing radioactivity on SPECT/CT and intraoperatively with the gamma probe. We checked the complete removal of the sentinel by inserting the gamma probe again into the dissection field to confirm the absence of residual activity in situ.

Postoperative PSA measurement after surgery was recommended at the first month, the third month and every three months of the first year, biannually in the second and third year and annually thereafter. PSA dynamics and additional treatment were registered in these cases. Biochemical recurrence (BCR) was defined as 2 consecutive PSA values ≥ 0.2 ng/mL. Biochemical progression-free survival (BPFS) was defined as the time from the operation to the day of BCR.

Exclusion criteria were a history of pelvic surgery, radiotherapy neo- or adjuvant treatment and incomplete pathological or follow-up data.

All patients were categorized into two groups: Group 1, traceable sLNs and Group 2, untraceable sLNs (no radiological or intraoperative signal). Medians, interquartile ranges and frequencies were used for descriptive statistics. The chi-square and *t* tests were used to compare pre- and postoperative characteristics between the following groups: age, PSA, cT, biopsy ISUP, percentage of positive cores, percentage of MSKCC nomogram, BMI, activation of ^99m^Tc-nanocolloid, pT, ISUP, R1, number of removed lymph nodes and LNI.

The following variables were evaluated as potential predictors of untraceable sLN in univariable analysis: age, preoperative PSA (10 vs. 10–20 vs. ≥20ng/mL), BMI (<26 vs. ≥26 kg/m^2^), ^99m^Tc-nanocolloid activity (<200 vs. ≥200 MBq), cT stage (cT1-cT2a vs. cT2b vs. ≥cT2c), biopsy ISUP, percentage of positive cores, PSMA PET/CT imaging, MSKCC nomogram estimate, pathological ISUP and pT stage (pT2 vs. pT3a vs. ≥pT3b).

BPFS rates were estimated using Kaplan–Meier analysis. All analyses were performed using SPSS software (version 23.0, SPSS). A *p*-value < 0.05 was considered statistically significant.

All patients provided written informed consent. The university’s ethical committee approved the prospective collection of the data (protocol no. BE-2-56).

## 3. Results

Table 1 summarizes the characteristics of our study cohort. A total of 53 patients who underwent RP with ePLND and sLD dissection were included in the ongoing prospective study (median age 63 years, median PSA 9.3 ng/mL). The study cohort was considered overweight with a median BMI of 26.1 kg/m^2^. Most had clinical stage ≥ cT2c (60.4%) and biopsy ISUP grade 2 (58.5%). The median preoperative MSKCC nomogram-predicted risk of LNI was 15%. The median injected activity of ^99m^Tc-nanocolloid was 169 MBq (IQR 127.5–303.5) and a median of 18 lymph nodes was removed per patient. After RP, pathological ISUP grade 2 remained the most frequent (54.7%) and almost half of the patients had ≥pT3a (49.1%). Histologically confirmed LNI was present in 10 patients (18.9%).

Overall, 41 patients (77.4%) exhibited traceable sLNs (Group 1), while untraceable sLNs (Group 2) were observed in 12 patients (22.6%). Most baseline clinical and pathological characteristics were well balanced between the traceable and untraceable sLN groups. Age and preoperative PSA did not differ significantly between groups (age, *p* = 0.07; PSA, *p* = 0.1). Preoperative MSKCC nomogram estimates were similar between groups (median 15% [IQR 9–23] overall; *p* = 0.4). The ^99m^Tc-nanocolloid activity (MBq) showed no significant intergroup differences (*p* = 0.9) nor did BMI (*p* = 0.2). Clinical stage distribution showed no statistically significant difference between groups (*p* values for comparisons not significant in the presented data). Biopsy ISUP and pathological ISUP distributions were comparable between groups. Pathologic stage (pT2 versus pT3/4) showed no significant between-group difference (*p* = 0.3). The number of removed lymph nodes was comparable between groups (median 18 nodes each; *p* = 0.8). R1 rates were similar between groups (approximately 29–33%; *p* = 0.8).

LNI was present in 10/53 patients overall (18.9%), and differed significantly between the two groups, with a higher proportion in Group 1 than in Group 2 (*p* = 0.016). Specifically, 24.4% of Group 1 had LNI versus 0% in Group 2. Among 10 patients with LNI, 8 had one pathological lymph node and 2 had two pathological lymph nodes. All metastatic deposits were micrometastases measuring from 1 to 8 mm and were considered to be as sLN, as proven by SPECT/CT and/or intraoperative gamma probe. None of these patients underwent PET/PSMA imaging preoperatively.

In univariable analyses, none of the predictors were associated with untraceable SN (*p* > 0.05) (Table 2).

**Figure 1:**
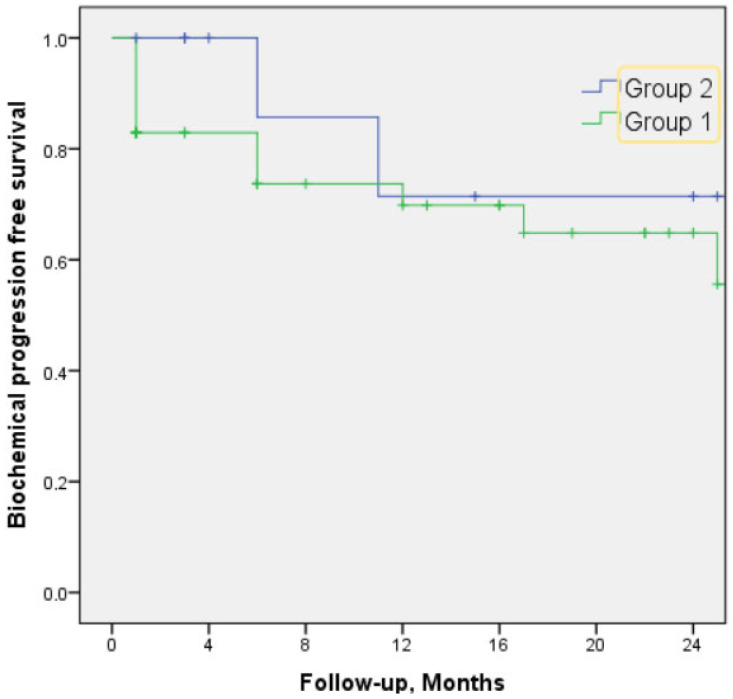
Biochemical progression-free survival.

The median time of follow-up after RP was 22 months (IQR 8–32). Over this time, 17/53 of men (32.1%) experienced BCR, overall, with higher observed events in Group 1 (15/41, 36.6%) than in Group 2 (2/12, 16.7%). The 24-month BPFS rate was 55.6% in Group 1 and 71.4% in Group 2; however, this difference did not reach statistical significance (*p* = 0.2) (Figure 1).

## 4. Discussion

The role of ePLND in PCa remains debated, as robust oncologic outcome data are not universally established. Nevertheless, ePLND is frequently performed for staging and to facilitate risk-adapted adjuvant strategies. The procedure is associated with longer operative time and a spectrum of morbidities, including vascular, nerve or ureteral injuries, postoperative thromboembolic events, lymphocele formation, longer hospital stay and adverse health-related quality of life [11,17]. Therefore, PLND remains among the most interesting topics for urologists. Given these considerations, sLN dissection has emerged as a rational step to refine nodal staging. Although widely adopted in melanoma, breast, vulvar and penile cancers, sLN dissection in PCa remains under investigation and is considered to be an experimental approach [2,7,13,14,18,19,20,21,22].

The first report of sLN dissection in prostate cancer appeared in 1999 by Wawroschek et al. [7], using ^99m^Tc-nanocolloid, with nodal metastases observed in 26.8% cases. In our prospective series, LNI was detected in 18.9% of cases, a rate compatible with existing literature. Importantly, LNI is influenced by both the extent of nodal dissection and preoperative factors. Daigle R et al. [23] compared ePLND vs. super-ePLND (sePLND), extending the dissection to include regions from the aortic bifurcation, common iliac and presacral areas. The groups were not heterogeneous; the sePLND group had worse preoperative parameters according to D’Amico criteria. The complication rate did not differ between the groups (*p* = 0.3). It should be noted that patients who underwent neoadjuvant treatment were not excluded from the study, and there were more patients in the sePLND group (0.7% vs. 10%). Despite the use of neoadjuvant therapy, postoperative pathological features were also worse in the sePLND group, with a substantially higher LNI rate (7.6% vs. 36.1%, *p* < 0.001) and greater median number of removed lymph nodes (14 (IQR 10–19) vs. 19 (14–27), *p* < 0.001).

Although we limited dissection to the traditional ePLND template in most patients and extended it beyond standard boundaries only in a minority with sLNs outside conventional areas, our median lymph node yield was competitive in both analyzed groups (18, IQR 13.5–22) and in line with other studies in which sLN dissection with ePLND was performed [17,18,21].

Meinhardt et al. [18] analyzed 35 participants who underwent laparoscopic RP with sLPND and ePLND using ^99m^Tc-nanocolloid for sLN detection with an intraoperative gamma probe. In three patients, no sLN was identified on scintigraphy, and in another three, scintigraphy was positive, but no activity was detected with the probe during surgery (6/35, 17.1%). These three patients had a time interval of >24 h between injection and surgery. All six patients underwent ePLND. However, the authors do not mention whether these patients had LNI. Following these events, they modified their protocol so that surgery was performed 4–24 h after injection (the scintigrams showed no change after 4 h). Similar cases of untraceable sLN have been reported in other studies [14,17,18,19,21]. Taking this info into account, in our study, all patients underwent surgery within 20 h after the injection of ^99m^Tc-nanocolloid.

CT scanning is used to exclude the presence of metastases and to refine clinical staging. Muteganya R et al. [20] reported that radiological criteria for metastatic lymph nodes include a short axis diameter > 1 cm for oval nodes and >0.8 cm for round nodes. Using such criteria, a meta-analysis found that, for the detection of positive nodes, CT has a pooled sensitivity of 42% and a specificity of 82%. However, in some cases, even larger metastases may be missed. Rousseau et al. [19] demonstrated that, when using the sentinel lymph node dissection technique, metastases with a diameter of up to 30 mm can be identified, and they were missed with a preoperative CT scan.

Our findings are consistent with the broader literature supporting sLN techniques as a means to improve nodal staging in clinically localized PCa. In our series, LNI was diagnosed in 10 out of 53 cases (18.9%). A total of eight patients had one pathological lymph node, and the remaining two patients had two pathological lymph nodes. All of these were micrometases, measuring 1–8 mm in diameter, and were considered sentinel lymph nodes, because they were identified using preoperative SPECT/CT and/or an intraoperative gamma probe. None of these patients underwent PSMA PET/CT imaging.

PSMA PET/CT imaging has specific size limitations for detecting lymph node metastases in prostate cancer patients, although its performance is superior to that of CT. Recent guidelines consider PSMA PET/CT a more accurate radiological tool for PCa staging, and it is anticipated that CT scans will be used less frequently in the future and largely replaced by PSMA PET/CT [2]. The spatial resolution limit of PSMA PET/CT appears to be around 3–5 mm (with a sensitivity of 54% for a 3 mm lymph node), and smaller metastases (≤2 mm) are generally below the detection threshold and often are untraceable [24]. Some authors propose that a negative PSMA PET/CT scan may be used to reduce unnecessary ePLND in PCa patients [25]. Hinselveld F.J. et al. [26] performed RP with sLN dissection and ePLND for patients who were preoperatively PSMA PET/CT-negative. A total of 6/31 patients (19%) had LNI, with a median metastasis diameter of 2 mm (IQR 1–3.8), and all metastases were located in sLN. Other authors reported similar findings [27]. These results suggest that although PSMA PET/CT is highly accurate, micrometastases may still be missed if ePLND is omitted in such patients.

Although sLN mapping provides good results, it should be noted that not all sLN are traceable during SPECT/CT or with an intraoperative gamma probe. The reported rate of untraceable sLN is up to 30% [13,14,17,19,20,21,22]. Theoretically, the may be several reasons for this. Inaccurate mapping techniques that utilize low-volume radioactive tracers or rely only on indocyanine green (ICG) or blue dye can fail to identify sLNs during the operation [28]. A large tumor burden (macrometastasis) and associated fibrosis may block the lymphatic pathway, altering drainage patterns and making it more challenging to detect sLNs. Previous surgeries, radiotherapy or inflammation can lead to scarring or lymphedema, obstructing lymphatic pathways and complicating sLN identification. Individual patient characteristics, such as obesity, may also affect lymphatic flow and the visibility of sLN detection [29].

It should be noted that none of the patients in our cohort had a history of pelvic surgery, radiotherapy, or neoadjuvant/adjuvant treatment. All patients underwent preoperative CT and bone scan; only cN0 cases were included in the final analysis to rule out macrometastasis. In our presented study, 12 out of 53 patients (22.6%) had untraceable sLN. Brenot-Rossi I. et al. [22] investigated this issue. None of the evaluated factors (age, ISUP grade, pathological stage) demonstrated statistical correlation with sLN detectability. The authors showed that increasing the dose of ^99m^Tc-nanocolloid from 60 MBq to 200 MBq reduced the rate of untraceable sLN to 7.1% (2/28 patients) with a statistically significant association (*p* < 0.034). However, their cohort showed more favorable postoperative pathological features compared with ours: only 12 patients (12%) with LNI and 2 of them were in the group with untraceable sLN. In our analysis, we also did not find any correlation with pathological parameters, nor did we observe any significant difference related to the dose of ^99m^Tc-nanocolloid (*p* > 0.05). All patients in Group 2 (with untraceable sLN) had no LNI, despite undergoing ePLND.

PSMA PET/CT became available relatively late at our institution. We supplemented our methodology by incorporating this test, and patients with PSMA PET/CT-positive results were excluded from the study. For this reason, only seven (13.2%) patients of the entire cohort underwent this imaging modality and all of them were PSMA PET/CT-negative. Two of the patients were in the untraceable sLN group. Our hypothesis is that if preoperative imaging- particularly PSMA PET/CT- is negative and sLN are untraceable, the risk of LNI is very low. Our cohort suggests that, under these circumstances, ePLND could have been omitted in approximately 23% of patients. Nevertheless, further studies are required to validate this approach.

This prospective study has several limitations. The relatively small sample, particularly in Group 2, limits the precision of effect estimates and the ability to detect statistically significant differences in outcomes between the groups. The second limitation is the short follow-up period after RP, which was only 22 months. During this time, 17 men (32.1%) experienced BCR, with a higher rate of events in Group 1 (15/41, 36.6%) compared to Group 2 (2/12, 16.7%), but there was no significant difference between the groups. Given the short follow-up, it is questionable whether this duration is sufficient to draw meaningful conclusions regarding oncological outcomes. Additionally, the single-center design may introduce selection bias and limit the general applicability of the findings. Nevertheless, since this is an ongoing prospective study, the number of patients will increase, as well as the follow-up duration, which may alter the final results in the future.

## 5. Conclusions

These are the first results from an ongoing prospective study. A proportion of prostate cancer patients lack radiologically or intraoperatively detectable sentinel lymph nodes. Notably, none of the patients with untraceable sLNs exhibited lymph node metastases following ePLND, while most baseline clinical and pathological characteristics were well balanced between both groups. These findings suggest that untraceable sentinel lymph nodes may be associated with an extremely low probability of nodal invasion and could serve as a criterion for safely omitting PLND in selected patients.

## Figures and Tables

**Table 1:** Patient characteristics.

Parameter	All *n* = 53	Gr1 41 (77.4)	Gr2 12 (22.6)	*p* Value
Age (years): median (IQR)	63 (57–68)	62 (57–66)	67.5 (59–69)	0.07
PSA (ng/mL): median (IQR)	9.3 (5.7–12.1)	8.7 (5–11.4)	11.3 (8.8–13.8)	0.1
Clinical stage: *n*, (%)				0.2
cT1	3 (5.6)	3 (7.3)	-	
cT2a	10 (18.9)	7 (17.1)	3 (25)	
cT2b	8 (15.1)	8 (16.5)	-	
cT2c	11 (20.8)	7 (17.1)	4 (33.3)	
cT3	21 (39.6)	16 (39)	1 (8.3)	
Biopsy ISUP: *n*, (%)				0.8
1	4 (7.5)	3 (7.3)	1 (8.3)	
2	31 (58.5)	24 (58.5)	7 (58.3)	
3	8 (15.1)	8 (19.5)	-	
4	9 (17)	6 (14.6)	3 (25)	
5	1 (1.9)	-	1 (8.3)	
% of positive biopsy: median (IQR)	62.5 (39.2–90.8)	62.5 (38.5–90.1)	64.6 (42.5–96.1)	0.7
PSMA PET/CT testing (yes): *n*, (%)	7 (13.2)	5 (12.2)	2 (16.7)	0.7
MSKCC preop. nomogram (%): median (IQR)	15 (9–23)	13 (9–23)	20 (9.5–26.8)	0.4
Pathological ISUP: *n*, (%)				0.8
1	1 (1.9)	1 (2.4)	-	
2	29 (54.7)	22 (53.7)	7 (58.3)	
3	10 (18.9)	7 (17.1)	3 (25)	
4	9 (17)	7 (17.1)	2 (16.7)	
5	4 (7.5)	4 (9.8)	-	
Pathologic stage: *n*, (%)				0.3
pT2	27 (50.9)	23 (56.1)	4 (33.3)	
pT3a	19 (35.8)	14 (34.1)	5 (41.7)	
pT3b-pT4	7 (13.2)	4 (9.8)	3 (25)	
No. of removed LN: median (IQR)	18 (13.5–22)	19 (12.5–22.5)	18 (16.25–20.5)	0.8
LNI: *n*, (%)	10 (18.9)	10 (24.4)	-	0.016
R1: *n*, (%)	16 (30.2)	12 (29.3)	4 (33.3)	0.8
Activity of ^99m^Tc-nanocolloid (MBq): median (IQR)	169 (127.5–303.5)	159 (125–303.5)	193 (133.5–303.3)	0.9
BMI kg/m^2^: median (IQR)	26.1 (25.4–27.5)	26.4 (25.5–27.6)	25.5 (24.9–27.2)	0.2
BCR: n, (%)	17 (32.1)	15 (36.6)	2 (16.7)	0.2

BCR—biochemical recurrence, BMI—body mass index, CT—computed tomography, IQR—interquartile range, ISUP—International Society of Urological Pathology, MSKCC—Memorial Sloan Kettering Cancer Center, LN—lymph nodes, LNI—lymph node invasion, PET—positron emission tomography, PSA—prostate-specific antigen, PSMA—prostate-specific membrane antigen, R1—positive surgical margin status.

**Table 2:** Univariable logistic regression analyses, using preoperative and postoperative features for predicting untraceable sLN.

	Univariable Analysis	
Predictors	Odds Ratio (95% CI)	*p* Value
Age	0.9 (0.8–1.01)	0.07
PSA ng/mL		
<10	Reference	
10–20	0.29 (0.07–1.18)	0.084
>20	1.15 (0.11–11.8)	0.9
BMI kg/m^2^		
<26	Reference	
≥26	2.4 (0.65–9)	0.2
^99m^Tc activation MBq		
<200	Reference	
≥200	0.7 (0.2–2.58)	0.6
Clinical stage		
T1–T2a	Reference	
T2b	0.65 (0.2–2.5)	0.9
≥T2c	0.76 (0.17–3.44)	0.72
Biopsy ISUP		
1	Reference	
2	1.1 (0.1–12.7)	0.91
3	0.9 (0.3–10.1)	0.9
4	0.67 (0.05–9.4)	0.77
5	0.6 (0.2–9.1)	0.7
% of positive cores	0.99 (0.97–1.02)	0.75
PET/PSMA		
No	Reference	0.7
Yes	0.7 (0.12–4.13)	
MSKCC nomogram	0.99 (0.95–1.04)	0.73
Pathological ISUP		
1	Reference	
2	1.3 (0.3–11.2)	0.87
3	1.2 (0.4–10.1)	0.56
4	0.72 (0.3–8.2)	0.7
5	0.62 (0.31–7.2)	0.73
Pathological stage		
T2	Reference	
T3a	0.49 (0.11–2.12)	0.34
≥T3b	0.23 (0.37–1.45)	0.12

CI—confidence interval.

## Data Availability

Data are not publicly available due to patient privacy and institutional ethical restrictions. De-identified data underlying the findings of this study may be made available from the corresponding author on reasonable request and subject to approval by the institutional ethics committee.
